# Office design as a risk factor for disability retirement: A prospective registry study of Norwegian employees

**DOI:** 10.5271/sjweh.3907

**Published:** 2020-12-16

**Authors:** Morten Birkeland Nielsen, Jan Shahid Emberland, Stein Knardahl

**Affiliations:** 1 National Institute of Occupational Health, Oslo, Norway

**Keywords:** cellular office, cost, health, open office, open-plan office, shared office, sickness absence, work ability, workplace architecture

## Abstract

**Objectives:**

This aim of this study was to (i) examine differences in risk of subsequent disability retirement between employees working in cellular, shared, and open-plan offices and (ii) determine the contribution of gender, skill-level, work ability, medically certified sickness absence, leadership position, and personality traits (extroversion, agreeableness, conscientiousness, neuroticism, and openness) as confounders.

**Methods:**

Survey data on predictor variables combined with official objective registry data on disability retirement and sickness absence were extracted from a large Norwegian occupational cohort of office workers (N=6779, 53.5% women). Questionnaire data included the respondents’ office designs, comparing cellular, shared, and open-plan offices, demographic characteristics, workability, and personality factors. Objective data on disability retirement and medically certified sickness absence were extracted from the sickness and disability benefit register of the Norwegian Labor and Welfare Administration.

**Results:**

In the final fully adjusted model, employees working in shared [hazard rato (HR) 1.52, 95% confidence interval (CI) 1.08–2.16] and open-plan (HR 1.95, 95% CI 1.31–2.90) offices had significantly higher risk of subsequent disability retirement compared to employees in cellular offices. Gender, work ability, medically certified sickness absence, and conscientiousness had independent direct effects on risk of disability retirement.

**Conclusion:**

This study shows that open and shared workspace designs have detrimental effects by increasing risk of disability retirement among office workers, even when taking other known predictive factors into account.

The move of office workers from cellular offices to shared or open-plan workspaces is a predominant trend in contemporary working life. Reducing space per employee – and thereby the costs of office space – is an obvious motivator for this change. It is also commonly assumed that open-plan office layouts will facilitate social interactions and communication, enhancing innovation and productivity (1). On the other hand, contrasting such assumptions about the potential benefits of open-plan workspaces, emerging evidence from both primary studies and systematic reviews indicate that open and shared office design layouts also have significant costs as manifested through increased health problems (2–4) and sickness absence rates (5–7) among employees. However, research on outcomes of office designs has been criticized. A recent scoping review of the literature argued that most previous studies were based on inadequate study designs (8). With a few notable exceptions (7, 9), previous studies of office design layouts have reported subjective evaluations of outcomes such as distractions, satisfaction, well-being, productivity, and sickness absence. Self-report measures may be influenced by extraneous factors like the design and context of the measurements and susceptible to method bias (10). Consequently, one may argue that cost estimates of subjective factors are prone to error and/or that costs of the subjective effects recorded so far are negligible. Hence, there is a need for studies of objectively measured outcomes of office designs.

In addition to the limitations in study designs, few studies have investigated potential confounding factors that can explain the associations between office designs and outcomes (8). For instance, as previous research has shown that levels of work ability and sickness absence disability retirement (11–13), work ability and sickness absence are likely to confound associations between office design and risk of disability retirement. Furthermore, office design encompasses a plethora of different layouts and principles. Open-plan offices may be based on fixed seating positions or shared activity-based seating positions, the space or area per employee may vary, and the availability of sound-attenuated cubicles or meeting rooms may differ, and so on. Such variations in office design layouts determine the nature and frequency of exposure to sounds, noise, and visual stimuli from other employees. An important aspect in this regard is that individual differences in perceptions and appraisals of such exposures may vary (14). Accordingly, previous findings suggest that age, gender, job status and role in the organization could influence individual satisfaction with, and reactions to, workspace design and layouts (15, 16).

The personality characteristics of employees may play an especially important role with regard to outcomes of office design but have received limited attention in past research. Drawing upon the five-factor model of personality (FFM) (17, 18) it seems reasonable to assume that certain individual traits may be particularly relevant with regard to how office designs affect the individual worker. In particular, due to the extrovert’s preference for activities that involve social interactions and group work, high levels of extroversion could be associated with a more positive appraisal of sharing space with other people. Neuroticism is another personality trait of the FFM that is likely to influence the outcomes of office designs. As neuroticism is associated with negativity, maladjustment, and problems handling stressors (19), high levels of neuroticism may be associated with lower tolerance for stimuli that are likely to be present in open workspaces.

Effects of the FFM personality traits in *combinations* with type of office design (cell, shared room, open-plan, and flex) on self-reported distractions, job satisfaction, and job performance were examined in 1205 Swedish employees (20). The findings showed that low levels of neuroticism were associated with *lower* levels of distraction, particularly among those working in *flex offices*, whereas both *agreeableness* and *openness to experience* were associated with *higher* levels of distraction among participants in *open-plan* compared to *cellular offices* (20). No interactions were found between extroversion and office type in relation to distraction, nor did the personality traits influence the impact of office designs on job satisfaction and performance.

Addressing the limitations of previous research, the present study will add to knowledge of the effects of workspace design by including objectively measured outcome data, ie, officially registered disability retirement, and examining the role of appropriate confounding variables. Disability retirement incurs large costs and knowledge about the causes is therefore a highly relevant and important outcome. In Norway, a country with a population of approximately 5.3 million people, about 369 500 persons received disability retirement compensations in 2018. The national cost for disability benefits was 86.3 billion kroner (about UK£8 billion; see www.regjeringen.no/no/aktuelt/folketrygdens-utgifter/id2613905). This is equivalent to about 2.4% of Norway’s gross domestic product (GDP) of 3536 billion kroner for 2018. Several previous studies have reported that psychosocial work factors contribute to early retirement due to disability (21). Considering that office design contributes to functioning, productivity, and health among employees (22), it seems reasonable to expect that office design is also a predictor for disability retirement. The two main objectives of the present study were to determine (i) associations between cellular, shared, and open-plan office designs and risk of subsequent disability retirement and (ii) whether the associations between office designs and risk of disability retirement among employees are influenced by gender, work ability, levels of medically certified registry based sickness absence, skill level, leadership position, and personality traits as reflected through the FFM of personality.

## Methods

### Study design

This study is a part of the research project: “The new workplace II: work factors, sickness absence, and exit from working life among Norwegian employees”. The study protocol provides a full description of the research project, procedures, and data material, including demographic information (23). The data material encompasses survey responses (questionnaire) linked with official registry data on medically certified sickness absence and disability benefits. Survey responses were collected from a large sample of adults employed in a full- or part-time position. Subjects were recruited from organizations in Norway that were contacted and offered to participate in the study. At the organizational level, this sampling procedure was based on a convenience approach with no pre-defined criteria for participation. All employees, excluding those on long-term sick leave, were mailed a letter with information about the survey, which explained the aims of the project and assured that responses would be treated confidentially in strict accordance with the general guidelines and specific license from the Norwegian Data Protection Authority. The survey was mainly web-based although about 15% of the participants completed a paper version due to limited access to computers at work. Type of survey response method (web versus paper) was not related to subsequent risk of disability retirement.

The organizations from which employees were recruited provided data on employees’ departmental affiliation, home address, and occupational title according to the Norwegian standard classification of the occupations (STYRK) – a system developed by Statistics Norway based on the International Classification of Occupation (ISCO-88). In return for participation in the project, the organizations received written reports and oral presentations of results to support management and personnel in the process of monitoring their work conditions.

### Ethical approval

The Regional Committees for Medical and Health Research Ethics (REC) in Norway approved this study, which has permission from the Norwegian Data Protection Authority and was conducted in accordance with the World Medical Association Declaration of Helsinki. All study participants provided their informed consent. When accessing the web-based questionnaire by a personal login code, informed consent had to be confirmed before responding to the questionnaire. The Norwegian Data Protection Authority and REC approved this consent procedure. Respondents were treated anonymously in the data analyses. Only respondents who actively (by response) permitted the linking of their answers to official registries were included in this study. For the respondents consenting to registry linkage, we had access to information on disability retirement compensation recorded in the Norwegian Labor and Welfare Administration (NAV) registry up to 1 January 2015.

### Respondents

From November 2004 to March 2014, organizations encompassing a total of 30 585 employees were invited to participate in the survey for the first time. At the time of invitation 28 883 subjects were aged 18–62 and eligible for disability retirement. Employees aged 62–66 may also receive disability pension but are additionally entitled to early statutory pension. Consequently, and as we did not have access to the statutory pension registry, subjects >62 years of age were excluded from the present study. Of the subjects eligible for disability pension only, 16 651 responded to any of the exposure measures in the questionnaire relevant to this study (response rate: 57.6%). Altogether 14 501 permitted linking their responses to official registry data on sickness absence and disability retirement from the Norwegian Labor and Welfare Service (acceptance rate: 87.1%). As the aim of this study was to examine the impact of office design on risk of disability retirement, only respondents that reported working in a cellular, shared, or open-plan office were retained for analyses. After removing respondents that did not work in an office, the final sample for this study comprised 6779 respondents.

### Questionnaire instruments

*Office design* was assessed with a single item question phrased: “Do you work….” (i) “alone in your own office”, (ii) “In a shared office with one or more colleagues”, (iii) “In an open-plan workspace”, (iv) “In a shop/service station, etc.”, (v) “Treatment institution”, or (vi) “Outdoors”. Respondents who reported alternatives iv–vi were not included in this study as they do not work in an office.

*Self-reported work ability* was assessed with a previously validated single item from the work ability index (WAI; 24). This item is phrased: “We assume that your work ability can be valued with 10 points at its best. How many points will you give your current work ability (0 means that you are unable to work at the moment)?” Responses were given on an 11-point scale ranging from 0 (“without ability to work”) to 10 (“work ability at its best”).

Information about *gender* and *leadership position* was assessed with single item questions. Response categories for leadership position were “no” and “yes”.

*The big-5 personality factors* were measured with a 15-item abbreviated version of the International Personality Item Pool (IPIP; 25) developed by Nielsen & Knardahl (26). The questionnaire measures extroversion, agreeableness, conscientiousness, neuroticism, and openness with three items for each subscale. Each item is rated on a 7-point Likert scale (from “very inaccurate” to “very accurate”). Due to limitations in other indicators such as Cronbach’s alpha, mean inter-item correlation between items has been suggested as the most adequate indicator of internal consistency in short personality markers (see 27–29). Briggs & Cheek (28) recommend an optimal range for the mean inter-item correlation of 0.2–0.4. In this study, all scales had internal consistency within the recommended range at both measurement points, thus indicating high reliability: Extroversion (0.40), agreeableness (0.35), conscientiousness (0.30), neuroticism (0.34), and openness (0.23).

### Registry data on skill-level, disability retirement and sickness absence

Information about employee skill-level were extracted from the employee registries of the participating organizations. Skill levels were determined by classification of occupation according to the International Standard for Classification of Education (ISCED). The skill level classification reflects the differences in education or the respective occupations. The levels were: 1=occupations that normally require education equivalent to a first or postgraduate university degree, or college exams based on a similar length of study (>16 years); 2=occupations that normally require 1–3 years of education at university or college (but not equivalent to the first university-level) (13–15 years); 3=occupations that normally require 1–3 years of secondary education (10–12 years); 4=occupations that require ≤9 years of primary education; and 5=unspecified (occupations in which the level of education may vary substantially). In cases where no information on occupational group (ISCO-88) had been provided by the subjects’ respective companies, missing values were substituted with self-reported skill level information (N=102).

Based on informed consent from participants, survey data were linked to the sickness and disability benefit NAV register by the unique 11-digit national identity number. The registers provide complete records of disability retirement that are compensated by the national insurance sickness benefit (30). All residents of Norway are members of the National Insurance Scheme. Residents aged 18–66 who have been a member of the National Insurance Scheme for at least three consecutive years before the onset of disease, illness, or injury are eligible for the disability pension scheme (31). A disability retirement is only granted to those with a physician-certified permanent reduction in the ability to work of minimum 50%. Time on sick-leave is not a criteria for disability retirement. Information about specific diagnoses were not available. Hence, the present study investigated all-cause disability retirement.

Information on official register-based medically certified sickness absence included complete registrations of all medically certified sickness absence 12 months prior to and 12 months after the survey. The current study focuses on absence prior to the survey, although findings on absence succeeding the survey also are presented. The current study had access to data on total number of days with medically certified absence but not the number of absence spells, duration of spells, or medical diagnosis.

### Statistical analysis

Data analysis was conducted with SPSS 23.0 (IBM, Armonk, NY, USA) and R version 3.2.2 (survival package). Scale variables (ie, personality indicators) were treated as continuous variables in the analyses. Hazard ratios (HR) and 95% confidence intervals (CI) were calculated with Cox regression analysis to determine the influence of office design on post-response risk of disability retirement. Cox regression (or proportional hazards regression) is a method for investigating the effect of several variables upon the time at which a specified event takes place. As recommended for studies in healthy populations (32), attained age (at censoring/ event) was the underlying time scale in these analyses rather than “time-on-study” (ie, years since baseline response). However, to address the impact of time on study, the length of the follow-up period was included as a covariate in the final fully adjusted regression model. The use of age as the time scale variable made age adjustment redundant in the Cox regressions. Missing data were excluded with listwise deletion. Gender, days with medically certified sickness absence 12 months prior to the survey, having a leadership position, work-ability, and skill level were included as covariates in all adjusted analyses. Because the last category of the skill-level variable was unspecified, reflecting varying degrees of educational attainment, the variable was treated as nominal in all analyses.

The analyses were performed in four steps. In step one, disability retirement was regressed on office design without taking into account confounding variables. In step 2, gender, medically certified sickness absence, self-reported work ability, skill-level, and leadership position were included as control variables. Step 3 included all of the aforementioned variables and added the personality markers extroversion, conscientiousness, agreeableness, openness, and neuroticism. Medically certified sickness absence and self-reported work ability were included as potential confounding variables since employees with low levels of work ability and/or high levels of absence may have been provided with separate cellular offices as a measure to prevent premature working life exit. Since the respondents participated at different time-points, the analyses were adjusted for the length of the follow-up period (before linkage to the registry data) in step 4.

Subjects were censored at the end of follow-up (1 January 2015) or earlier in case of death, emigration, or reaching the eligible age for early statutory pension (62 years). Mean follow-up time for the respondents was 6.5 (SD 2.7; range 1.0–10.1) years. We examined the proportional hazards assumption by the testing of non-zero slopes and plotting scaled Schoenfeld residuals. No violation of the assumption was detected (P>0.05).

## Results

### Prevalence rates and descriptive findings

The majority of the sample conducted their work in a cellular office (56.5%), while 26.2% worked in a shared office, and 17.3% worked in an open-plan office. A total of 226 persons (3.3%) became recipients of disability retirement in the course of the study period. Demographic characteristics for the sample and bivariate associations between demographic characteristics and disability retirement are presented in table 1. Mean age in the sample was 47.77 (SD 9.68; range: 20–62) years. Mean self-reported work ability (range 0–10) was 8.70 (SD 1.49). Female respondents were significantly more likely to receive disability retirement compared to male respondents. Risk of disability retirement was highest among respondents in occupations normally requiring 13–15 years of education whereas the lowest risk was found among respondents in occupations normally requiring >16 years of education and in occupations in which the level of required education varies substantially. Non-leaders had significantly higher risk of disability retirement than respondents in leadership positions.

**Table 1:** Demographic characteristics for study sample (N=6779). Mean age 47.77 (SD 9.68) years; mean work ability 8.70 (SD 1.49).[ISCO=International Classification of Occupation.]

Variable	N	%	Prevalence disability retirement (%)	Group difference disability retirement (χ^2^)	Effect size differences
Gender				49.62 a	0.086
Male	3069	45.3	1.6		
emale	3626	53.5	4.7	
Days with sickness absence 12 months prior to survey				127.49 a	0.107
0	4168	65.9	2.8		
1–7	619	9.8	3.3		
8–14	406	6.4	3.6		
15–21	237	3.7	2.3		
22–28	114	1.8	3.7		
>28	784	12.4	11.8		
ISCO skill level				10.24 b	0.003
1	2647	39.1	3.0		
2	2029	29.9	3.8		
3	1142	16.9	4.3		
4	130	1.9	2.3		
5	827	12.2	2.1		
Leadership position				2.92 b	0.021
No	5071	75.6	3.5		
Yes	1638	24.4	2.6		

a P<0.001.

b P<0.05.

Differences in the study variables between the office design categories are displayed in tables 2 and 3. Although statistical differences were found between the office designs with regard to age of the respondents, length of follow-up period, and work ability, the effect sizes show that that the actual differences were very small. There were significant, but small, differences in the prevalence of subsequent disability retirement between the three office designs (χ^2^=6.17; df=6779/2; P<0.05) as respondents in shared offices (4.1%) and open-plan offices (3.7%) had higher rates than respondents in cellular offices (2.9%). As for personality traits, a one-way ANOVA indicated significant differences between the respondents in the different office designs concerning scores on openness (F=5.32; df=6415/2; P<0.01) and neuroticism (F=4.68; df=6415/2; P<0.01), but not for scores on extroversion, agreeableness, or conscientiousness. A Bonferroni post hoc test showed that respondents in cellular offices exhibited higher scores on openness compared to respondents in shared offices, and lower scores on neuroticism compared to respondents in shared and open-plan offices. However, estimates of effect sizes indicated that the actual differences in both neuroticism (partial eta^2^ 0.001) and openness (partial eta^2^ 0.002) between the office designs were very small.

**Table 2:** Differences in continuous study variables between office designs.

Variable	Office design			Group difference	Effect size differences
	
	Cellular (N=3828)	Shared (N=1781)	Open-plan (N=1170)		
			
	Mean (SD)	Mean (SD)	Mean (SD)	F	
Age	49.26 (9.30)	46.31 (9.82)	44.99 (9.7)	120.18^a^	0.034
Follow-up period	6.37 (2.62)	6.69 (2.90)	6.45 (2.57)	8.60^a^	0.003
Work ability	8.75 (1.44)	8.62 (1.65)	8.66 (1.37)	20.38 b	0.001
Extroversion	4.86 (1.28)	4.92 (1.27)	4.85 (1.28)	1.50	0.000
Conscientiousness	5.94 (0.90)	5.96 (0.95)	5.90 (0.96)	1.25	0.000
Agreeableness	5.40 (0.90)	5.42 (0.94)	5.36 (0.90)	1.66	0.001
Openness	5.54 (0.92)	5.46 (0.94)	5.51 (0.91)	5.32 c	0.002
Neuroticism	3.55 (1.24)	3.64 (1.23)	3.66 (1.23)	4.68 c	0.001

a P<0.001.

b P<0.05.

c P<0.01.

**Table 3:** Differences in categorical study variables between the office designs. [ISCO=International Classification of Occupation.]

Variable	Office design			Group difference	Effect size differences

	Cellular (N=3828)	Shared (N=1781)	Open-plan (N=1170)		
			
	%	%	%	χ^2^	
Disability retirement				6.17 a	0.023
No	97.1	95.9	96.3		
Yes	2.9	4.1	3.7		
Gender				8.92 a	0.036
Male	47.2	45.1	42.4		
Female	52.8	54.9	57.6		
Days with sickness absence 12 months prior to survey				39.96 b	0.027
0	68.0	61.4	66.8		
1–7	8.5	11.6	12.3		
8–14	6.7	6.4	4.9		
15–21	3.5	4.4	4.1		
22–28	1.7	2.3	1.7		
>28	11.7	13.8	10.2		
ISCO skill level				803.87 b	0.053
1	46.9	23.9	36.6		
2	22.4	39.1	40.7		
3	11.7	30.2	13.3		
4	1.6	2.8	1.8		
5	17.5	4.0	7.5		
Leadership position				164.55 b	0.15
No	69.7	82.7	84.1		
Yes	30.3	17.3	15.9		

a P<0.05.

b P<0.001.

### Office design impact on risk of disability retirement

Findings from the Cox regression analysis with attained age (at censoring/event) as the underlying time scale are presented in table 4. Analyses were conducted in four steps with additional confounding variables at each subsequent step. Office design had a significant main effect on disability retirement in step 1 of the regression. Respondents working in shared offices (HR 1.86, 95% CI 1.38–2.50) and open-plan offices (HR 1.87, 95% CI 1.31–2.67) had a significantly higher risk of disability retirement when compared to respondents in cellular offices. The association between office design and disability retirement remained significant after adjusting for gender, self-reported work ability, days with sickness absence during the 12 months before the survey, skill-level, and leadership position in the second step. High work ability reduced the risk of disability retirement (HR 0.79, 95% CI 0.75–.84), whereas female gender (HR 2.49, 95% CI 1.79–3.46) and having >28 days of sickness absence during the year prior to the survey (HR 2.41, 95% CI 1.73–3.36) increased the risk of disability retirement.

**Table 4:** Associations between office designs and registry based disability retirement (Cox regressions; N=6779). [HR=hazard ratio; CI=confidence interval; ISCO=International Classification of Occupation.]

Variable	Step 1 (N=6778) a		Step 2 (N=5498) b		Step 3 (N=5106) c		Step 4 (N=5106) d	
			
	HR	95% CI	HR	95% CI	HR	95% CI	HR	95% CI
Office design								
Cellular (reference)								
Shared	1.86	1.38–2.50	1.46	1.06–2.01	1.57	1.11–2.21	1.58	1.18–2.22
Open-plan	1.87	1.31–2.67	1.75	1.20–2.54	1.92	1.30–2.86	1.93	1.30–2.87
Work ability			0.79	0.75–0.84	0.79	0.75–0.84	0.79	0.74 - 0.84
Days with sickness absence 12 months prior to survey								
0 (reference)								
1–7			1.13	0.67–1.89	1.17	0.67–2.01	1.17	0.69–2.00
8–14			1.24	0.70–2.22	1.42	0.80–2.55	1.41	0.78–2.54
15–21			0.68	0.28–1.68	0.83	0.34–2.06	0.82	0.33–2.02
22–28			0.54	0.13–2.21	0.59	0.14–2.41	0.60	0.15–2.46
>28			2.41	1.73–3.36	2.33	1.62–3.35	2.36	1.64–3.38
Gender								
Males (reference)			2.49	1.79–3.46	2.21	1.55–3.16	2.21	1.55–3.15
ISCO skill level								
1 (reference)								
2			1.36	0.98–1.89	1.26	0.88–1.79	1.12	0.77–1.63
3			1.21	0.82–1.76	1.18	0.79–1.78	1.12	0.74–1.70
4			1.64	0.50–5.36	1.35	0.32–5.57	1.13	0.27–4.83
5			0.77	0.41–1.45	0.69	0.35–1.36	0.69	0.35–1.35
Leadership responsibility			1.04	0.69–1.57	1.04	0.68–1.60	1.05	0.68–1.11
Extroversion					1.02	0.91–1.14	1.02	0.91–1.15
Conscientiousness					0.85	0.73–0.98	0.85	0.73–0.99
Agreeableness					1.13	0.95–1.34	1.12	0.96–1.36
Openness					1.06	0.91–1.24	1.06	0.94–1.33
Neuroticism					1.13	0.99–1.27	1.12	0.99–1.27
Length of follow-up period							1.06	0.99–1.12

a Step 1. Crude, unadjusted, model.

b Step 2. Adjusted for work ability, sickness absence 12 months prior to survey, gender, skill-level, and leadership position

c Step 3. Personality traits added as additional confounders

d Step 4. Length of follow-up period added as additional confounder.

The five personality markers were added to the regression in the third step. Conscientiousness was the only personality variable that had a significant relation with risk of disability retirement (HR 0.85, 95% CI 0.73–0.98). The coefficient shows that higher scores on the conscientiousness variable were associated with lower risk of disability retirement.

**Figure 1:**
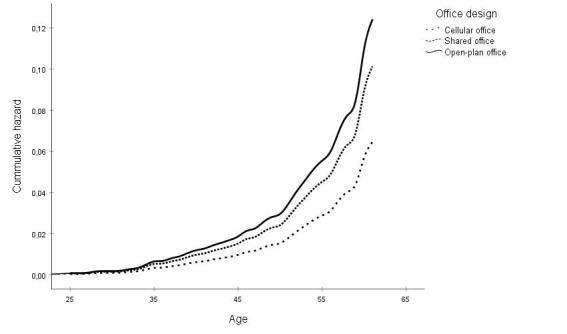
Associations between office design and subsequent risk of disability retirement (adjusted for all covariates in study). Age as time-scale variable.

In the fourth and final step, the analyses were adjusted for the length of the follow-up period between survey response and linking to registry data. Length of follow-up period was not associated with risk of disability retirement (HR 1.06; 95% CI 0.99–1.12). In this final model, the established associations between office designs and risk disability retirement remained significant as employees in shared offices (HR 1.58, 95% CI 1.12–2.22) and open-plan offices (HR 1.93, 95% CI 1.30–2.87) exhibited higher risk of disability retirement compared to employees in cellular offices. Work ability, sickness absence, gender, and low level of conscientiousness remained significant predictors of disability retirement. A graphical presentation of the associations between office design and disability retirement from the fully adjusted model are shown in figure 1.

Step 2–4 of the Cox-regression was reanalyzed using days of absence 12 months *succeeding* the survey, instead of prior to the survey, as an indicator of medically certified sickness absence. The findings were consistent with the main analysis. In the fully adjusted model (step 4), respondents working in shared (HR 1.47, 95% CI 1.04–2.06) and open-plan offices (HR 1.73, 95% CI 1.16–2.56) had significantly higher risk of disability retirement when compared to respondents in cellular offices. Having >28 days of sickness absence during the 12 months succeeding the survey (HR 3.76, 95% CI 2.71–5.21) increased the risk of disability retirement.

## Discussion

Based on official registry data on disability retirement from a large Norwegian occupational cohort of office workers, the present prospective study showed that working in a shared or open-plan office is associated with an increased risk of early retirement from work due to disability when compared to employees working in cellular offices. Findings from adjusted analyses showed that the risk of disability retirement was independent of the respondents’ gender, skill-level, number of days with medically certified sickness absence, leadership position, self-reported work ability, personality traits, and length of follow-up period between survey response and linking to registry data. Secondary findings from this study indicate that work ability, female gender, >28 days with sickness absence during a 12 month period, and lower levels of conscientiousness have independent main effects on subsequent risk of disability retirement.

Having established office design as a risk factor for disability retirement, it is important to provide mechanisms that can explain this association. Previous research on the effects of office design on health outcomes has pointed to an increased risk of infectious diseases as a possible explanation for why employees in shared and open-plan offices report more health problems and higher sickness absence rates (7). However, it is unlikely that transmission of viruses should lead to permanent disability and early retirement. Hence, alternative explanations for how and why type of office design may lead to disability retirement seem warranted. In the following, we will highlight two plausible and potentially interconnected explanations that may be a topic for upcoming research. First, sharing office workspace implies coping with distractions from noise and behavior of other persons (33–35). Humans tend to pay attention to speech and sharing an office may pose added demands for concentration and tax the tolerance for distractions. Indeed, based on questionnaires and room acoustic measurements in 21 offices, it has been found that distracting background speech largely explains the overall perception of noise in that the less the speech intelligibility, the lower the share of employees disturbed by noise (34). Constant noise can make you tired and lead to a sense of sensory overload, even a three-hour exposure to simulated office noise can lead to increased urinary adrenaline levels (35). Finally, self-reported frequent exposure to disturbing noise at work is associated with increased risk of long-term sickness absence among office workers (33). Consequently, effects of noise in open offices spaces may result in more tiredness, fatigue, and health complaints including headache and mental distress (36, 37). The combination of these health problems may eventually facilitate exit from working life. In support of this hypothesis, frequent self-reported exposure to disturbing noise at work has been found to be associated with increased risk of long-term sickness absence among office workers (33).

A second explanation why open workspaces may increase the disability risk is that such office architecture may compromise the need for privacy, ie, not being constantly observed or listened to by others (9). Privacy is a fundamental human need (9, 38) and diminished privacy may inflict dissonance and distress. Reduced opportunities for privacy may substantially affect perceptions of “control” ie, the possibilities for a person to influence what happens in their work environment. Notably, a recent systematic review concluded that job control was the most consistent work-related predictor of disability retirement (21).

Both the above explanations highlight health problems as the mechanism that explains how office design may influence risk of disability retirement. Hence, a limitation of our study is that we did not have access to the specific diagnoses for the disability pension grant. The current study is therefore unable to inform whether the higher risk of disability retirement when working in shared or open workspaces mainly are due to somatic or mental health issues. Such information would have strengthened the study and should therefore be included in upcoming replications.

The magnitude of the association between office design and disability retirement remained significant after accounting for the effects of gender, self-reported work ability, medically certified sickness absence, skill-level, leadership position, personality, and length of follow-up period. Both female gender, medically certified sickness absence, and work ability contributed independently to the disability retirement-risk rate – indicating that future research of the impacts of office design on the disability risk should account for these factors. The substantive effect of gender corroborates findings of past disability retirement studies (39, 40). The impact of self-reported work ability also confirms previous findings indicating that individuals’ negative evaluation of their work ability predict subsequent exit from working life due to disability (11). Hypothetically, work ability could confound the association between office layout and disability retirement since employees with lower levels of work ability may be given a cell office as a measure of workplace accommodation or adaptation. However, it should also be noted that work ability, as well as sickness absence, may be operating as a mediators; type of office design should arguably have the potential of affecting level of work ability, which in turn, affects the risk of premature retirement. Thus, the adjustment of work ability and sickness absence in our analyses may have partialled out some proportion of the substantive effect of office design on the disability retirement-risk.

Although it has been argued that the outcomes of office design vary due to individual differences among employees (14), our findings showed that the magnitude of the association between office design and disability retirement was not influenced by personality traits (41, 42). Based on common sense, one might presume that extroversion, which includes preferences for social settings and a tendency to be outgoing, would be beneficial in shared or open-plan offices. At the other end of the extroversion–introversion spectrum, one might presume that introversion, which includes preferences for solitary experiences, would be negative in open workspaces. However, in line with a previous study on the role of personality in outcomes of office design (20), our findings did not suggest any impact of level of extroversion on the association between office design layout and risk disability retirement.

Although the examined personality traits had no impact on the association between office designs and disability retirement, we found an important direct relation between the conscientiousness trait and disability retirement as respondents with high scores on the trait had lower risk of disability retirement. Conscientiousness is defined as the relatively stable pattern of individual differences in the tendencies to follow socially prescribed norms for impulse control, be goal-directed, planful, delay gratification, and follow norms and rules (41). Evidence indicate that people with higher scores on the conscientiousness trait are healthier and live longer lives (42), and it is therefore not surprising that higher scores on conscientiousness were associated with lower risk of disability retirement in this study. It should also be noted that the neuroticism trait had a close to significant relation with risk of disability retirement. Neuroticism is a trait that predisposes to health problems and is therefore also likely to be a risk factor for disability (43, 44). Due to the uncertainty of estimates in a single sample study, the role of neuroticism, in addition to conscientiousness, with regard to disability should therefore be further examined in upcoming studies.

### Strengths and limitations

The prospective study design, large sample size, and use of official registry data to assess disability retirement are strengths of this study. The fact that the average work ability scores in the sample were high and that employees on long-term sick-leave were not invited to the survey suggest that the baseline population was healthy. There were no major changes in national regulations of disability benefits in the survey period that could have influenced our findings. The general economy of Norway was excellent throughout the follow-up period with low levels of unemployment compared to most other countries. It is likely that the financial situation of a country influences the health and work ability of workers, as well as the national welfare benefits such as disability retirement schemes. As Norway is a relatively wealthy country where the welfare programs are highly prioritized by the government, direct comparisons with countries that have other types of welfare arrangements should therefore be done with caution.

Although the survey had a response rate in line with the estimated average for organizational surveys (45), altogether 52% of invited respondents did not participate in the questionnaire survey. While the sample was large, the non-random recruitment of participating organizations limits the external validity of the findings. However, it should be noted that probability sampling at the individual level was conducted as all employees in the participating organizations were invited to survey participation (46).

The survey data of this study were collected between 2004 and 2014. Hence, at the individual level, the follow-up period varies between the respondents. Compared to respondents with a shorter follow-up period, it is likely that respondents with a longer follow-up may have experienced changes in the design of the workplace or in their perceptions of the workplace. The follow-up period was therefore considered in the analyses by including a time-scale variable and adjusting for the length of the period. The associations between office design and disability retirement remained significant even after this adjustment.

The question about office design had only three response categories. Employees may have access to several kind of office solutions for their work (eg, flex-offices) and thereby select the working place according to the task at hand. A more refined indicator could have provided more detailed information about the actual office design and whether respondents used more than one type of office solution during their workday. On the other hand, due to the relatively low incidence of disability retirement cases during the follow-up period, a more fine-grained measure with several response categories would require a larger sample size in order to detect differences. Still, future research could extend our result by adding further information about office design and the physical work environment, such as distraction due to noise (33, 34). With regard to the indicator of office design, the phrasing of the response categories for “shared office” and “open-plan” offices may have led to some overlap as respondents who work in small open-plan offices may have considered this as a shared cellular office.

The respondents’ skill-level was used as an indicator of work task. This is a relatively coarse way of categorizing work tasks and a more fine-grained categorization with more specific information about the tasks may have led to other results. The exposure data on office type should be valid since the subject reported his or her present office type at baseline. Work ability was assessed with a previously validated single item from the work ability index. The employed FFM personality instrument has been psychometrically tested in a previous study (26). However, it should be noted that this condensed version of the original inventory has its limitations by not providing information about the sub-facets of each trait. Nevertheless the complete 240-item Revised Neuroticism– Extroversion–Openness Personality Inventory (NEO-PI) (47), may not suitable for incorporation in investigations primarily addressing work environment aspects.

### Comparison with other studies

To our knowledge, this is the first study of the impact of office design layout on risk of disability retirement. Previous studies have reported that working in shared offices or open-plan offices increases the risk of sickness absence compared to individual cellular-type offices (5–7). However, only the study by Nielsen & Knardahl (7), which utilized an overlapping sample to the current study, was based on registry data. Hence, the findings of the present study extend previous research by showing that working in an open space design is a risk factor for subsequent disability retirement.

While a previous study found that personality characteristics influence the association between office designs and outcomes (14), we found no confounding effects of personality traits in the current study. However, the association between office design and disability retirement was somewhat attenuated when adjusting for gender, medically certified sickness absence, and work ability, thus pointing to these variables as important confounders. The finding that high levels of sickness absence are associated with increased risk of disability retirement is in line with previous research (12, 13).

### Concluding remarks

Early exit from working life due to disability retirement may lead to a poorer quality of life, loss of social identity, and mental complaint (48). Knowledge about predictors of retirement due to disability is therefore important. Our findings from a Norwegian setting indicate that open and shared workspace designs could have negative effects in the form of increased risk of employee disability retirement, even when taking other risk factors for disability retirement into account. To reduce the risk of disability retirement, organizations and employers may benefit from addressing well-known challenges inherent in open workspaces such as auditory and visual noise (49), reduced privacy (1), and reduced communication and interaction (9). Providing employees with the opportunity to use cellular offices may be one way of dealing with these challenges. Future research should determine the mechanism that can explain how office design increase the risk of disability retirement and also investigate the generalizability of our findings to other countries and settings. In order to extend this study, future research should apply a more refined measure of office design that allows for investigating different kinds of open-plan offices, such as flex offices and activity-based working.
